# Development of a Magnetic Bead-Based Method for Specific Detection of *Enterococcus faecalis* Using C-Terminal Domain of ECP3 Phage Endolysin

**DOI:** 10.4014/jmb.2302.02033

**Published:** 2023-04-28

**Authors:** Yoon-Jung Choi, Shukho Kim, Jungmin Kim

**Affiliations:** 1 Department of Biomedical Sciences, The Graduate School, Kyungpook National University, Daegu 41944, Republic of Korea; 2 Department of Microbiology, School of Medicine, Kyungpook National University, Daegu 41944, Republic of Korea

**Keywords:** Detection of bacteria, *Enterococcus faecalis*, cell binding domain, bacteriophage endolysin, magnetic beads

## Abstract

Bacteriophage endolysins are peptidoglycan hydrolases composed of cell binding domain (CBD) and an enzymatically active domain. A phage endolysin CBD can be used for detecting bacteria owing to its high specificity and sensitivity toward the bacterial cell wall. We aimed to develop a method for detection of *Enterococcus faecalis* using an endolysin CBD. The gene encoding the CBD of ECP3 phage endolysin was cloned into the *Escherichia coli* expression vector pET21a. A recombinant protein with a C-terminal 6-His-tag (CBD) was expressed and purified using a His-trap column. CBD was adsorbed onto epoxy magnetic beads (eMBs). The bacterial species specificity and sensitivity of bacterial binding to CBD–eMB complexes were determined using the bacterial colony counting from the magnetic separations after the binding reaction between bacteria and CBD–eMB complexes. *E. faecalis* could bind to CBD–eMB complexes, but other bacteria (such as *Enterococcus faecium*, *Staphylococcus aureus*, *Escherichia coli*, *Acinetobacter baumannii*, *Streptococcus mutans*, and *Porphyromonas gingivalis*) could not. *E. faecalis* cells were fixed onto CBD–eMB complexes within 1 h, and >78% of viable *E. faecalis* cells were recovered. The *E. faecalis* recovery ratio was not affected by the other bacterial species. The detection limit of the CBD–eMB complex for *E. faecalis* was >17 CFU/ml. We developed a simple method for the specific detection of *E. faecalis* using bacteriophage endolysin CBD and MBs. This is the first study to determine that the C-terminal region of ECP3 phage endolysin is a highly specific binding site for *E. faecalis* among other bacterial species.

## Introduction

Enterococci are major pathogens that are responsible for various hospital-acquired infections, such as endocarditis, neonatal sepsis, bacteremia, catheter-associated urinary tract infections, and meningitis [1-4]. Enterococci have also been reported to promote inflammation in periodontal disease [5]. Patients with periodontitis, an advanced form of periodontal disease, are categorized into three stages of severity: mild, moderate, and severe [6]. Anaerobic or weakly aerobic bacteria, such as *Porphyromonas gingivalis*, are often considered causative agents of periodontitis [7]. However, we previously reported a significant presence of *Enterococcus faecalis* in samples from patients with severe periodontitis [8]. Enterococci can develop resistance to antimicrobials; for example, vancomycin-resistant enterococci often cause difficult-to-treat hospital-acquired infections [9].

Bacteriophages (phages) are viruses that infect bacteria. These viruses inject their genome into the host bacterial cell, use enzymes for phage genome replication, transcription, and translation to produce phage progeny, and eventually burst out from the host cell before infecting new hosts [10]. Endolysins play an important role in the phage life cycle by enzymatically breaking down peptidoglycan in the bacterial cell wall [11, 12]. Some of the enzymes involved in this process exhibit modular architectures with catalytic domain(s) and a cell binding domain (CBD) [13, 14]. Innovative approaches for the selective binding and isolation of bacterial cells can be developed by exploiting the CBD properties of phage endolysin [15, 16].

We previously reported the isolation of a novel *E. faecalis* phage ECP3, named ‘vB_EfaM-ECP3’, according to current rules [17]. ECP3 belongs to the *Myoviridae* family and possesses a contractile tail. It specifically lyses *E. faecalis* but does not lyse other bacterial species, including *E. faecium*. The phage genome is a double-stranded linear DNA, which is 145,518 bp long and contains 220 open reading frames. The ECP3 genome was found to share 97% sequence identity with 90% of the *Myoviridae* phage PhiEF24C genome [18, 19]. The endolysin (N-acetylmuramoyl-L-alanine amidase) of ECP3 is identical to that of PhiEF24C and also identical to Lys70 except for one single amino acid in the amino acid sequence. The C-terminal domain of the two-domain structured endolysin was found to be a binding module to *E. faecalis*, but the binding specificity was not thoroughly examined with various bacterial species [20, 21].

Herein, we developed a simple and convenient method for the highly specific detection of *E. faecalis* using ECP3 endolysin recombinant CBD protein and magnetic beads (MBs) for the first time, which are used as carriers for the recovery of *E. faecalis* cells. This method enables the rapid detection of *E. faecalis* in a specimen such as dental plaques without the need for conventional repetitive culture processes.

## Materials and Methods

### Bacterial Strains

The bacterial type strains, including *E. faecalis* ATCC 29212, *E. coli* ATCC 25922, *Acinetobacter baumannii* ATCC 17978, *Staphylococcus aureus* ATCC 25923, and *Streptococcus mutans* ATCC 25175, were purchased for this study. Further, 1 *E. faecium* isolate, 1 *P. gingivalis* isolate, and 14 *E. faecalis* clinical isolates were obtained from the National Culture Collection for Pathogens (NCCP), South Korea. The bacteria were cultured at 37°C for 24–48 h in Mueller–Hinton broth (MHB; BD Difco, USA), Mueller–Hinton agar (MHA; BD Difco, USA), or Blood agar plate (BAP; Kisan Bio, Korea), as appropriate.

### Construction of CBD and CBD–Green Fluorescent Protein (GFP) Fusion Proteins Expression Vectors

The gene encoding the CBD of ECP3 lysin was amplified via PCR using the following primers: NdeI-ECPcbd (5¢-GGGAATTCCATATGGGTATGGACGTAGACGAAGTTGTACG-3¢) and XhoI-ECPcbd (5¢-ATCCGCTCGAGT CTGTTATACCATGGTGC-3¢). The amplified PCR product (357 bp) was purified, digested with NdeI and XhoI restriction enzymes (the target sequences are underlined in each primer sequence), and ligated into the pET21a expression vector (Novagen, USA), which was also digested using the same enzymes, resulting in the construction of pECPcbd (5,730 bp).

To visualize the CBD, enhanced GFP (eGFP) was fused with CBD using overlap PCR as follows. The CBD-encoding region was amplified via PCR (CBD-f: 5¢-GGGAATTCCATATGGGTATGGACGTAGACGAAGTTG TACG-3¢, CBD-r: 5¢-CCCTTGCTCACCATTCTGTTATACCATGGTGCATTTTTATTCCATTCT-3¢), and the eGFP-encoding gene was amplified using pEGFP (Clontech, USA) via PCR (eGFP-f: 5¢-CATGGTATAACA GAATGGTGAGCAAGGGCGAG-3¢, eGFP-r: 5¢-ATCCGCTCGAGCTTGTACAGCTCGTCCATGCCG-3¢). The amplified DNA products (1,102 bp) were digested with NdeI/XhoI and ligated into the pET21a+ vector as described above to construct pECPcbd–GFP (6,447 bp). The ligation products (ECPcbd and ECPcbd–GFP) were introduced into the *E. coli* BL21 (DE3) strain, and each transformant was selected on Luria–Bertani agar plates containing ampicillin (150 μg/ml). The resulting plasmids, pECPcbd and pECPcbd–GFP, facilitated the expression of recombinant proteins with a C-terminal 6-histidine tag (-LE-HHHHHH) under the control of the T7 promoter. The plasmids were further purified and verified via sequencing.

### Recombinant Protein Expression and Purification

Protein purification of CBD and CBD–GFP was performed as described previously [22]. Briefly, the *E. coli* BL21 (DE3) strain was transformed with pECPcbd and pECPcbd–GFP, and the proteins were induced by adding isopropyl β-D-1-thiogalactopyranoside (final concentration, 0.1 mM) for 18 h at 18°C. Bacterial cells were harvested and resuspended in lysis buffer (50 mM Tris–HCl, pH 7.5; 200 mM NaCl; and 0.008% β-mercaptoethanol) and then sonicated in an ice slurry. After centrifugation at 4°C, the supernatants were loaded onto a His-trap column (GE Healthcare, USA) that was installed in a fast protein liquid chromatography AKTA Prime PLUS chromatography system (Pharmacia, USA). The purified fractions were collected and analyzed using sodium dodecyl sulphate-polyacrylamide gel electrophoresis (SDS-PAGE) and western blot analysis with an anti-His-tag monoclonal antibody. The fractions of CBD with a His-tag (14.8 kDa) and CBD–GFP with a His-tag (41.7 kDa) were confirmed. These fractions were dialyzed in phosphate-buffered saline (PBS, pH 7.4) using Slide-A-Lyzer Mini Dialysis Device (10 kDa molecular weight cut-off, Thermo Scientific, USA) to remove any residual imidazole and replace the buffer. The protein concentration was measured using Pierce BCA Protein Assay Kit (Thermo Fisher Scientific, USA).

### Coating of MBs with Recombinant Proteins

Epoxy MBs (5.54 × 10^9^ beads/g, eMBs, Bioneear, Korea) were suspended in diethylene glycol dimethyl ether (Junsei Chemical, Japan) at a final concentration of 30 mg/ml, as recommended by the manufacturer. The beads (400 μl) were washed twice with 800 μl of Dulbecco’s PBS (DPBS) and resuspended in 100 μl of DPBS and 200 μl of 3M (NH_4_)_2_SO_4_ (pH 7.4). Then, 100 μl of CBD or CBD–GFP (1 mg/ml) was added, and the mixtures were incubated in an overhead rotator for 16 h at 12 rpm and 4°C. The mixtures were then incubated at 22°C for 6 h. Residual epoxy groups were blocked by washing the beads four times with DPBS–bovine serum albumin (BSA)(DPBS containing 0.1% BSA, pH 7.4). The CBD- or CBD–GFP-coated beads (2.0 × 10^9^ beads/ml) were stored at 4°C in DPBS–BSA buffer.

### Detection and Harvesting of *E. faecalis*

The CBD–eMB (1.66 × 10^8^ eMBs/ml) solution (100 μl) was mixed with 100 μl of a sample having bacterial suspension. The mixtures were then incubated in an overhead rotator for 1 h at 10 rpm and an ambient temperature. Then, CBD–eMB complexes were harvested using a magnetic bar, washed 4 times in 100 μl of DPBS, resuspended in 100 μl of DPBS, vortexed vigorously, and spread onto an MHA plate. The plate was incubated at 37°C for 20 h, and the number of bacterial colonies was counted.

### Measurements of Recovery Ratios

The number of bacterial cells released from CBD–eMB–bacterial cell complexes and that in the supernatant were determined by plating the samples in duplicate. The recovery ratios were calculated as follows: recovery ratio = (CFU from CBD–eMB–bacterial cell complexes) / initial input bacterial CFU × 100. All experimental measurements were presented as the mean values with standard deviations obtained from at least six replicates. Statistical analyses were performed using unpaired *t*-test, with a significance (α) level of 0.001.

### Confocal Microscopy and Scanning Electron Microscopy (SEM)

*E. faecalis* cells were stained with Biotium BactoView Live Red fluorescent dye BactoView Live Red fluorescent dye (Biotium, USA) to visualize the bacteria attached to CBD–GFP–eMB. Then, *E. faecalis* cells (10^9^ CFU) were suspended in PBS, centrifuged at 6,000 rpm, and washed twice. The dye was added to the bacterial samples at a final concentration of 1× and incubated at 37°C for 30 min in the dark. After mixing 100 μl of *E. faecalis* solution, which was stained with fluorescent red dye, with 100 μl of CBD–eMB–GFP solution, the sample was incubated for 60 min on a rotary stirrer at 10 rpm and room temperature under dark conditions. Subsequently, 10 μl of the sample was added onto a glass slide and after fixing the sample, it was examined under a confocal fluorescence microscope (LSM5, Zeiss, USA).

To observe the surface structures of CBD–eMB and CBD–eMB–bacteria complexes, 15 μl of the sample was added onto a glass slide and dehydrated through a series of ethanol washes (20%, 50%, 70%, and absolute ethanol) for 30 min. The samples were coated with gold and photographed using a field-emission scanning electron microscope (FE-SEM; Hitachi S-4300, Hitachi, Japan).

### Determination of the Optimal Conditions for CBD–eMB Binding to *E. faecalis*

To determine the optimal conditions for obtaining the best sensitivity and specificity of CBD–eMB binding to *E. faecalis*, CBD–eMB and *E. faecalis* solutions were mixed and incubated for different periods. The number of eMBs ranged from 3.32 × 10^6^ to 1.66 × 10^7^, and the incubation times ranged from 10 to 120 min. The eMBs were mixed with the bacterial suspensions, and the tubes were incubated on a rotary shaker at 12 rpm. After magnetic separation, the beads were resuspended in 100 μl of DPBS. The entire mixture was plated and incubated, and the cells were enumerated.

### Determination of the Sensitivity and Specificity of the CBD–eMB Complex for Detecting *E. faecalis*

In order to determine sensitivity of the CBD-eMB based method, 100 μl of CBD–eMB solution was prepared as described above; then, 100 μl of different concentrations of *E. faecalis* (from 5.4 × 10 to 5.4 × 10^9^ CFU) was mixed with 100 μl of DPBS by vortexing for 20 s, followed by incubation for 1 h at 12 rpm. The detection limit (sensitivity) was determined by observing bacterial colonies on MBs in cultured plates.

To examine *E. faecalis*-specificity of the CBD-eMB method, The CBD–eMB complex was incubated with different bacterial species (clinical isolates of *E. faecium*, *E. coli*, *A. baumannii*, *S. aureus*, *S. mutans*, and *P. gingivalis*) independently or mixed together. To confirm whether only *E. faecalis* was detected, six bacterial strains were mixed (Mix 6) at the same concentration (10^5^ bacterial cells in 100 μl) and incubated under the same conditions as described above. Fourteen clinical isolates of *E. faecalis* obtained from NCCP were also tested independently to determine the specificity of CBD–eMB complex.

### Statistical Analysis

One-way analysis of variance (ANOVA) followed by one-way Tukey’s test for all pairwise comparisons (95%confidence interval) was performed. The data are presented as the means with standard deviations. A *p*-value < 0.001 was considered statistically significant.

## Results

### Structural Analysis *in silico*

**Fig. 1:**
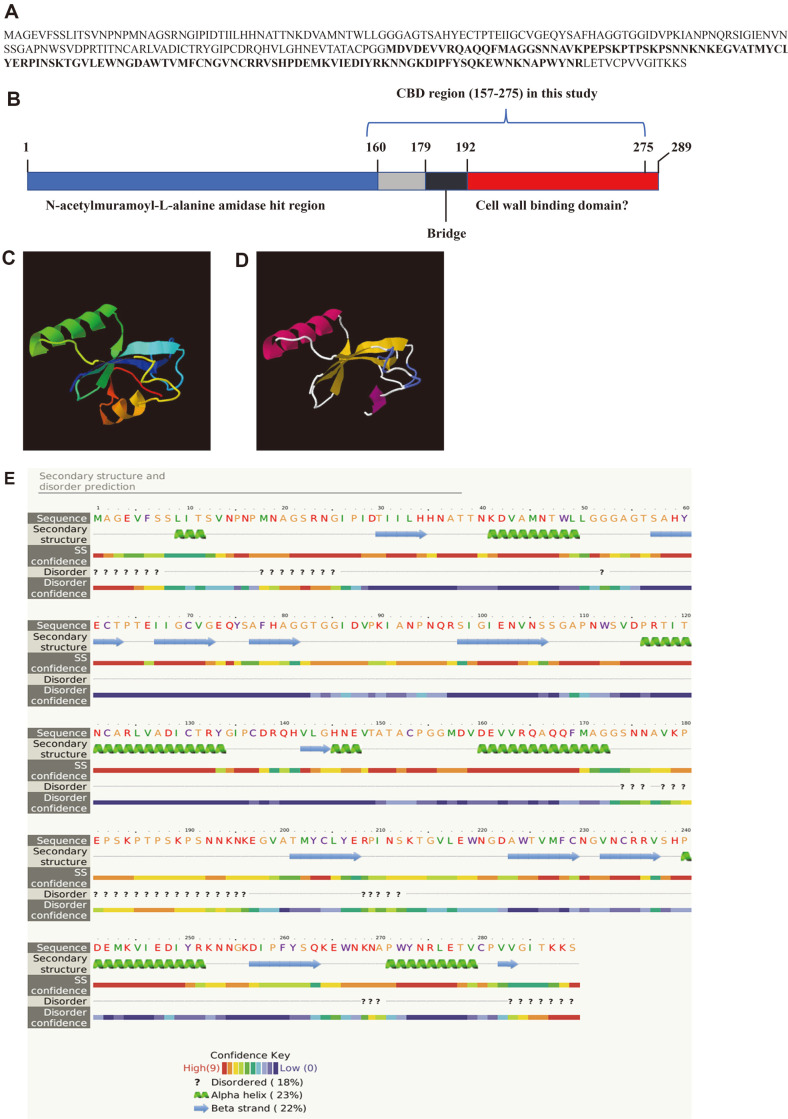
Amino acid sequence and predicted three-dimensional protein structure of LysECP3 endolysin and cell binding domain (CBD) protein. The prediction analysis of LysECP3 was performed using Phyre2. (**A**) Amino acid sequence of LysECP3. The bold letters represent the CBD region used in this study. (**B**) A schematic diagram of LysECP3. The blue region represents an enzymatically active domain (N-acetylmuramoyl-L-alanine amidase), and the red region represents a possible CBD. The CBD protein was generated with 119 amino acids (157th–275th). (**C**) The model was colored from the N terminus to C terminus of LysECP3, indicating the N-acetylmuramoyl-L-alanine amidase domain (from red α-helix to yellow region) and the C-terminal region (from green α-helix to blue and purple β-sheet). (**D**) CBD protein showing an α-helix (red) and β-sheet (yellow). (**E**) Predicted α-helixes, β-sheets, and disordered regions were indicated as color codes by confidence level (highest confidence: red; lowest confidence: purple).

A previous study used genomic DNA sequence and revealed that LysECP3, the endolysin of phage ECP3, comprises 289 amino acids and harbors an N-acetylmuramoyl-L-alanine amidase domain (26^th^–160^th^ amino acid) (Fig. 1A) [17]. According to PhiEF24C and Lys70 studies, the C-terminal domain of LysECP3 was expected to be a binding module to *E. faecalis* [20, 21].

The protein structure of LysECP3 was determined *in silico* using Phyre2 with 100% confidence in the model, in which hydrolases (lys170 CBD and lysgh15 amidase-2 domain) were used as templates (Fig. 1C) [23]. The predicted protein model showed the presence of a β-sheet between two α-helixes. The constructed CBD contained an α-helix and β-sheet without the enzymatically active domain (EAD) (Fig. 1D). The region extending from 179^th^ to 192^nd^ amino acids represented a disoriented region (a region that lacks a fixed or ordered tertiary structure), which might be a bridge or linker between the EAD and possible CBD (Figs. 1B and 1E), followed by three β-strands, one α-helix, one β-strand, and one α-helix (Fig. 1E). The CBD constructed in this study had a bridge region with no final α-helix (Fig. 1B).

### Purification of CBD and CBD–GFP Fusion Proteins

CBD (13.7 kDa) and CBD–GFP (41.7 kDa) were overexpressed and purified. The expected size of CBD proteins was observed in a single band in the SDS-PAGE gel, but CBD–GFP proteins were purified with some additional small-sized proteins (Fig. S1A). Western blot analysis revealed the expected bands of CBD and CBD–GFP proteins (Fig. S1B); therefore, we used these purified proteins for further experiments. The final concentrations of CBD and CBD–GFP proteins were 1.03 mg/ml (total 16 ml) and 2.18 mg/ml (total 16 ml), respectively.

### Specific Binding Analysis of CBD Protein

**Fig. 2:**
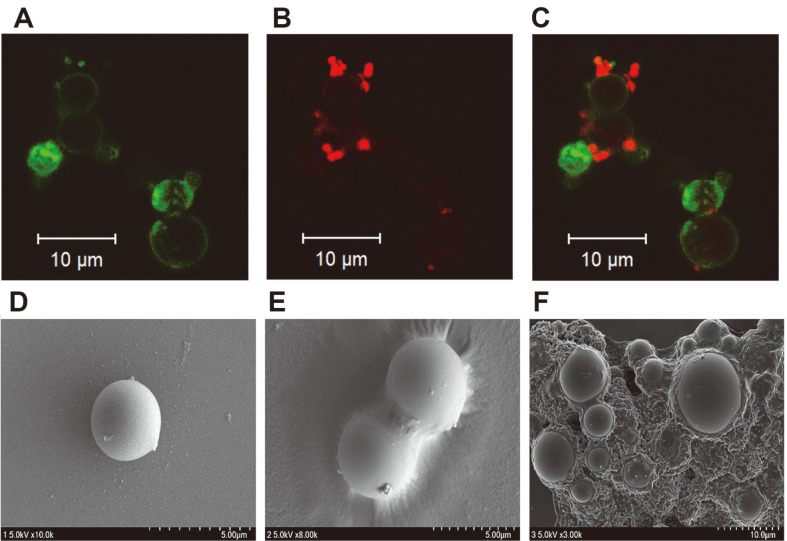
Images of *Enterrococcus faecalis* ATCC 29212 bound to recombinant protein-epoxy magnetic bead (eMB) complexes were obtained using confocal fluorescence microscopy and scanning electron microscopy (SEM). (**A-C**) Confocal fluorescence microscopy images of CBD–green fluorescent protein (GFP) fusion protein–eMB complex. (**A**) CBD–GFP–eMB complex bound to fluorescein isothiocyanate (FITC), green emission detected at 520 nm; (**B**) *E. faecalis* bound to CBD–GFP–eMB complex Cy 3, red emission detected at 675 nm; (**C**) overlap of (**A**) and (**B**). (**D–F**) SEM images of the CBD–eMB complex. (**D**) eMB; (**E**) CBD–eMB complex; (**F**) *E. faecalis* bound to CBD–eMB complex.

The recombinant CBD–GFP fusion proteins were coated on eMBs, as verified by green fluorescence under a confocal fluorescence microscope (Fig. 2A). After incubation with *E. faecalis* ATCC 29212, red fluorescent-stained bacteria were observed (Fig. 2B), which were bound to green fluorescent eMBs (Fig. 2C). This indicates that *E. faecalis* binds to CBD–GFP-coated eMBs. *E. faecalis* did not bind to GFP- or BSA-coated eMBs (data not shown).

Furthermore, the CBD protein was coated onto eMBs, as verified using matrix-assisted laser desorption/ionization time-of-flight mass spectrometry (MALDI–TOF MS) and FE-SEM. The mass spectra generated by the CBD protein (Fig. S2A) and CBD–eMB complex (Fig. S2B) were precisely matched with small and large peaks at 10,653 and 11,041 *m/z*, respectively (Fig. S2C), indicating that the CBD protein was coated onto the surface of eMBs. SEM analysis revealed intact eMBs with smooth surfaces (Fig. 2D). The CBD–eMB complex showed a soft-covered surface of eMBs (Fig. 2E). After incubation with *E. faecalis* ATCC 29212, bacteria were tightly bound to CBD–eMB complexes and appeared as a bacterial biofilm (Fig. 2F). These results demonstrated the strong binding of CBD toward *E. faecalis*. *E. faecalis* did not bind to GFP- or BSA-coated eMBs (data not shown).

### Sensitivity and Specificity of CBD-Coated eMBs for the Detection of *E. faecalis*

**Fig. 3:**
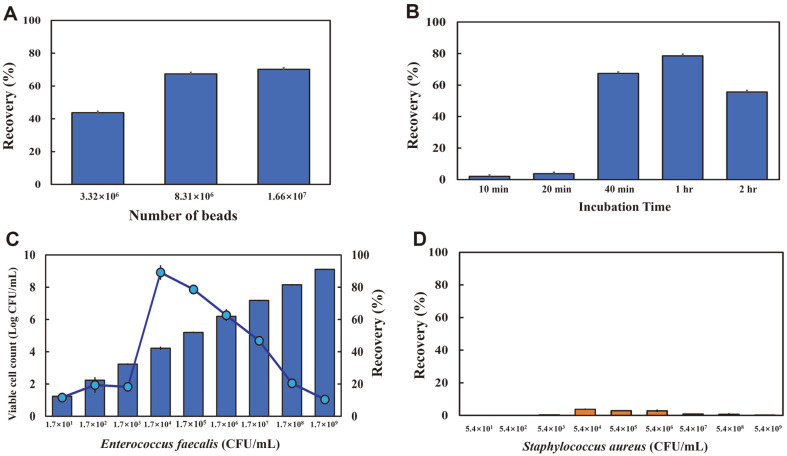
Optimization of binding conditions between *Enterococcus faecalis* and the CBD–eMB complex. (**A**) Quantitative binding conditions (number of CBD–eMB complexes ranged from 3.32 × 10^6^ to 1.66 × 10^7^, *E. faecalis* ATCC 29212 at 10^5^ CFU, incubation at room temperature for 1 h); (**B**) Incubation times (1.66 × 10^7^ epoxy magnetic beads, *E. faecalis* ATCC 29212 at 1.70 × 10^5^ CFU); (**C**) *E. faecalis* ATCC 29212 detection limit at the optimized binding condition (1.66 × 10^7^ CBD–eMB complexes, incubation at room temperature for 1 h); (**D**) No specificity of *S. aureus* ATCC 25923 at the optimized binding condition.

To optimize the binding between *E. faecalis* and CBD–eMB complexes, different numbers of CBD–eMB beads were analyzed under various incubation times. Three different quantities of MBs (3.32 × 10^6^, 8.31 × 10^6^, and 1.66 × 10^7^) were incubated with *E. faecalis* ATCC 29212 (1.4 × 10^5^ CFU/ml) for 1 h. Of the three incubation quantities, 1.66 × 10^7^ CBD–eMB yielded the highest recovery ratio (approximately 76%). The other quantities of CBD–eMB beads, *i.e.*, 3.32 × 10^6^ and 8.31 × 10^6^, yielded recovery ratios of approximately 44% and 69%, respectively (Fig. 3A, Table 1). The solutions were serially washed 4 times, wherein the number of CFUs dramatically decreased after the first wash, indicating that the binding strength of *E. faecalis* toward CBD–eMB complex was sufficient to resist the wash force (Table 1).

Further, different incubation times (10 min, 20 min, 40 min, 1 h, and 2 h) were used for the binding reactions with the same number of CBD–eMB beads (1.66 × 10^7^). Of the five incubation times, the 1-h incubation yielded the highest recovery ratio (approximately 79%). The other incubation times, *i.e.*, 10 min, 20 min, 40 min, and 2 h, yielded recovery ratios of approximately 3%, 37%, 67%, and 56%, respectively (Fig. 3B, Table 1). Regarding the number of CFUs during the washes, the 1-h incubation allowed the *E. faecalis* cell–CBD–eMB complex to be tightly bound, showing minimum CFU even after the first wash (0.42% of input bacteria). However, the *E. faecalis* cell–CBD–eMB complex became loose during the 2-h incubation and showed a greater number of CFUs in the first wash than that during the 1-h incubation (Table 1). The subsequent experiments were performed with 1.66 × 10^7^ CBD–eMB complexes with 1-h incubation.

The detection limit (sensitivity) of CBD–eMB complexes for *E. faecalis* was 1.70 × 10 CFU/ml (Fig. 3C). When *E. faecalis* concentrations ranging from 1.70 × 10 to 1.70 × 10^9^ CFU/ml were analyzed, the recovery ratio of *E. faecalis* ranged from 89.16% to 10.51% (Fig. 3C). The highest recovery ratio was observed at *E. faecalis* concentration of 1.70 × 10^4^ CFU/mL in the presence of 1.66 × 10^7^ beads. In contrast, the recovery ratio of *S. aureus* was <4% with CBD–eMB complexes (Fig. 3D).

**Fig. 4:**
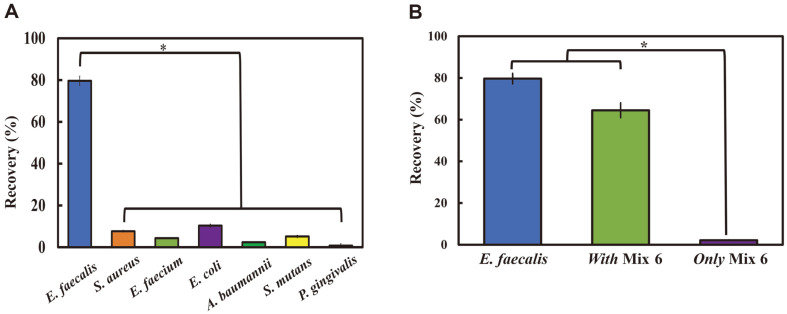
*Enterococcus faecalis* specificity of the CBD-eMB complex. (**A**) Bacterial recovery ratios of 7 bacterial species, respectively. (**B**) Bacterial recovery ratios of mixed bacterial solutions. With Mix 6 (*Enterococcus faecalis* plus *Staphylococcus aureus*, *Enterococcus faecium*, *Escherichia coli*, *Acinetobacter baumannii*, *Streptococcus mutans*, and *Porphyromonas gingivalis*), and only Mix 6 (six other bacterial species without *E. faecalis*). *p < 0.001 showing statistical significance.

We also determined whether bacteria other than *E. faecalis* (*S. aureus*, *E. faecium*, *E. coli*, *A. baumannii*, *S. mutans*, and *P. gingivalis*) can adhere to CBD–eMB complexes and found that only *E. faecalis* showed high recovery ratios (79.66% for *E. faecalis*, 7.71% for *S. aureus*, 4.38% for *E. faecium*, 10.38% for *E. coli*, 2.42% for *A. baumannii*, 5.2%for *S. mutans*, and 0.78% for *P. gingivalis*; Fig. 4A). Moreover, the recovery ratio of *E. faecalis* was maintained at 67.47% even after mixing with other bacteria (Fig. 4B). These results indicated that CBD–eMB complexes have high specificity to *E. faecalis* among tested bacterial species.

**Fig. 5:**
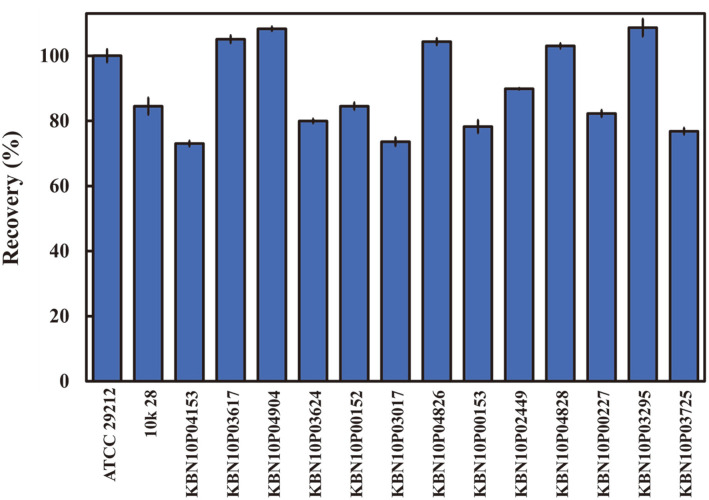
Recovery ratios of various clinical isolates of *Enterococcus faecalis*.

Various clinical isolates of *E. faecalis* were also analyzed for their ability to specifically bind to CBD–eMB complexes. The recovery ratio of *E. faecalis* clinical isolates was from 73% (KBN10P04153) to 108.6% (KBN10P03295)(Fig. 5). These recovery ratios were not significantly different from that of the standard *E. faecalis* strain ATCC 29212 (set to 100%, 2.82 × 10^2^ CFU/ml; Fig. 5). The detailed numerical raw data are presented in Supplemental Table 1.

## Discussion

The protein structure of endolysin is globular or modular. Most endolysins from phage that infect Gram-positive bacteria are modular proteins with enzymatic activity domain(s) and/or cell binding domains. LysECP3 only differs by a single amino acid substitution from the endolysin of phage F170/08 (Lys170), which has been characterized regarding its structure and function [20, 21]. However, to the best of our knowledge, our study is the first to test the binding specificity and sensitivity of the CBD protein on various bacterial species and clinical isolates of *E. faecalis*.

In this study, the CBD of LysECP3 could specifically bind to *E. faecalis* cell wall with high specificity. This finding indicated that CBD can be used as a biological probe to detect *E. faecalis* in clinical samples. The detection of *E. faecalis* using CBD–eMB complexes yielded high recovery ratios (approximately 90%). CBD–eMB complexes showed a statistically significant binding toward *E. faecalis* across varying ranges of bacterial concentration.

We revealed the unique properties of phage endolysin CBD in terms of the attachment and immobilization of *E. faecalis* cells onto solid surfaces. Purified recombinant CBD proteins were coated onto eMBs, which enabled the highly efficient capture and recovery of *E. faecalis* cells from suspensions by the magnetic separation. Xu *et al*. reported that the binding domain of Lys170 which is identical to LysECP3 formed tetramer revealed by protein crystallization study [20]. So, we believe that the purified CBD was gathered to be tetramers and attached to eMBs.

We also evaluated the applicability of different types of MBs for CBD proteins. MBs used to be coated with appropriate functional group which can be combined with DNA, RNA, proteins, or specific cells. Further, the combination of Ni-nitrilotriacetic acid (Ni-NTA) and MB was analyzed. The Ni-NTA MBs were heterogeneous, relatively large, and exhibited Ni-NTA ligands for affinity coating. The advantage of Ni-NTA MBs is their convenient coating procedure, high sedimentation ratio, and low surface/volume ratio; however, their unstable CBD binding precluded their further use for the isolation of whole bacterial cells at low concentrations (unpublished data). In contrast, eMBs are small, hydrophilic, polystyrene-coated particles whose specific surface chemistry allows for covalent protein immobilization [24]. These particles offer further advantages, including a low sedimentation ratio (increased availability of the coated surface), high stability, and high surface/volume ratio [25].

Our study demonstrated that phage proteins can be used as recognition molecules for the detection of bacteria. The receptor binding proteins, endolysin CBDs, and even whole phages are strong candidates for use as specific detection tools [26, 27]. The CBDs of phage endolysins have been successfully used to detect various gram-positive bacteria, such as *Listeria* spp. [28], *Bacillus cereus* [29], and *Clostridium tyrobutyricum* [30]. Antibodies, which have previously been used as specific binders, may exhibit cross reactivity and cause protein instability; moreover, the procedures for their preparation are expensive [31]. Therefore, the smaller and more stable CBDs offer advantages as specific binders. We used protein-coated MBs in this study, but other technical tools can be used; for example, biosensors and quantum dots labeling.

Clinical diagnosis of bacterial infections depends on various culture-independent diagnostic tests, such as nucleic acid amplification or ELISA-based antigen detection, MALDI–TOF MS, and whole-genome sequencing [32, 33]. These approaches may enable automated, reproducible, and easier detection of pathogen while allowing sensitive detection of nonculturable organisms and polymicrobial infections (multiplex detection). However, the specificity of these approaches may be affected by the detection of closely related nontarget species, which may lead to false-positive results [32, 34]. When *E. faecalis* was mixed with six other bacterial species, the recovery ratio was slightly lower than that of *E. faecalis* alone, indicating that *E. faecalis* was specifically recovered. In contrast, the recovery ratio of the mixture of six other bacterial species without *E. faecalis* was extremely low (<5%). When we analyzed 14 different clinical isolates of *E. faecalis*, the CBD–eMB complex could detect all clinical isolates. The detection limit of this complex was 10 CFU/ml for *E. faecalis* ATCC 29213 and 10^3^ CFU/ml for the clinical isolates of *E. faecalis*.

The development of rapid and reliable methods to detect pathogens is critical for the prevention and treatment of bacterial infections [35]. Culture-based diagnosis remains the gold standard in clinical microbiology; however, culturing of pathogens typically requires several days to weeks depending on the bacteria and can therefore be time- and labor-intensive [36, 37]. Early identification of pathogens is important to ensure that patients receive optimal antibiotic treatment in clinical settings [38]. Some rapid detection methods of *E. faecalis* have been developed using recombinase polymerase amplification, integrated with a lateral flow assay [39, 40] or real-time PCR [41], which are DNA sequence-based methods. Our CBD-magnetic bead-based method can detect and harvest live bacteria from samples, which can be further characterized bacteriologically.

*E. faecalis* was rapidly and accurately detected using a method based on the combination of a bacteriophage-derived recombinant protein with MBs in this study. The CBD of ECP3 phage endolysin is suitable for pathogen immobilization and capture required for diagnostic procedures. The yield of CBD protein is relatively high in one-step purification and the protein can rapidly and specifically bind to bacterial cells with a high sensitivity [15, 42-44].

In conclusion, we developed an efficient method for the specific detection of *E. faecalis* using a CBD protein from an *Enterococcus* phage endolysin and protein-coated MBs. This detection method offers a rapid and simple alternative to other diagnostic methods based on light microscopy, bacterial culture, and genetics. We believe that our findings will improve our understanding of the rapid, sensitive, and selective detection of *E. faecalis*.

## Supplemental Materials

Supplementary data for this paper are available on-line only at http://jmb.or.kr.

## Figures and Tables

**Table 1:** Optimization of the binding reaction between CBD-eMB and *Enterococcus faecalis*.

Incubation time and the number of magnetic beads		Attached CBD quantity (μg)	Initial CFU for addition (CFU/ml)	Attached CFU to BSA-eMB (CFU/ml)	Attached CFU to CBD-eMB (CFU/ml)	^1^Recovery (%)	CFU in Supernatant (%)	CFU in Wash 1 (%)	CFU in Wash 2 (%)	CFU in Wash 3 (%)	CFU in Wash 4 (%)
1 h	3.32 × 10^6^	100	1.45 × 10^5^	8.80 × 10^2^	6.39 × 10^4^	43.75	23.66	15.31	13.03	4.99	3.54
			± 0.413	± 0.124	± 0.041	± 1.837	± 2.339	± 3.375	± 2.510	± 3.402	± 0.994
	8.31 × 10^6^		1.41 × 10^5^	1.13 × 10^3^	9.81 × 10^4^	69.28	6.64	11.32	6.06	4.43	3.32
			± 0.388	± 0.124	± 0.097	± 4.861	± 1.446	± 2.301	± 1.112	± 2.214	± 1.341
	1.66 × 10^7^		1.47 × 10^5^	2.60 × 10^3^	1.13 × 10^5^	76.39	8.44	11.16	1.12	1.53	0.30
			± 0.065	± 0.124	± 0.165	± 0.694	± 1.446	± 1.163	± 0.894	± 0.679	± 2.877
10 m	1.66 × 10^7^	100	1.31 × 10^5^	5.74 × 10^2^	4.28 × 10^3^	2.85	62.21	22.44	6.62	4.31	0.42
			± 0.156	± 0.178	± 0.188	± 1.715	± 2.356	± 2.794	± 0.930	± 1.773	± 3.019
20 m			1.43 × 10^5^	8.83 × 10^2^	5.36 × 10^4^	37.11	29.65	24.20	6.62	1.57	0.72
			± 0.154	± 0.045	± 0.182	± 0.147	± 2.846	± 1.147	± 4.474	± 2.154	± 0.550
40 m			1.46 × 10^5^	2.24 × 10^3^	9.92 × 10^4^	67.36	21.03	9.11	1.58	0.33	0.07
			± 0.234	± 0.128	± 0.854	± 4.861	± 1.446	± 4.145	± 1.104	± 3.384	± 0.688
1 h			1.49 × 10^5^	2.68 × 10^3^	1.18 × 10^5^	78.77	19.66	0.42	0.42	0.02	0.01
			± 0.065	± 0.265	± 0.481	± 2.733	± 0.246	± 4.041	± 0.561	± 0.754	± 0.198
2 h			1.34 × 10^5^	1.42 × 10^3^	7.54 × 10^4^	55.64	26.57	12.54	0.66	2.16	1.42
			± 0.175	± 0.345	± 0.369	± 3.008	± 1.565	± 3.242	± 0.390	± 1.881	± 3.240

^1^Recovery (%) =((CFU from CBD–eMB–bacterial cell complexes) / initial input bacterial CFU) × 100.

## References

[1] Fernández-Guerrero ML, Verdejo C, Azofra J, de Górgolas M (1995). Hospital-acquired infectious endocarditis not associated with cardiac surgery: an emerging problem. Clin. Infect. Dis..

[2] Kotsanas D, Tan K, Scott C, Baade B, Cheng MHL, Tan ZV (2019). A nonclonal outbreak of vancomycin-sensitive *Enterococcus faecalis* bacteremia in a neonatal intensive care unit. Infect. Control Hosp. Epidemiol..

[3] Montalbán-López M, Cebrián R, Galera R, Mingorance L, Martín-Platero AM, Valdivia E (2020). Synergy of the bacteriocin AS-48 and antibiotics against uropathogenic enterococci. Antibiotics.

[4] Wang JS, Muzevich K, Edmond MB, Bearman G, Stevens MP (2014). Central nervous system infections due to vancomycin-resistant enterococci: case series and review of the literature. Int. J. Infect. Dis..

[5] Bhardwaj SB, Mehta M, Sood S, Sharma J (2017). Biofilm formation by drug resistant enterococci isolates obtained from chronic periodontitis patients. J. Clin. Diagn. Res..

[6] Graetz C, Mann L, Krois J, Sälzer S, Kahl M, Springer C (2019). Comparison of periodontitis patients' classification in the 2018 versus 1999 classification. J. Clin. Periodontol..

[7] Ardila CM, Bedoya-García JA (2020). Antimicrobial resistance of *Aggregatibacter actinomycetemcomitans*, *Porphyromonas gingivalis* and *Tannerella forsythia* in periodontitis patients. J. Glob. Antimicrob. Resist..

[8] Yoon DL, Kim S, Song H, Kim YG, Lee JM, Kim J (2015). Detection of bacterial species in chronic periodontitis tissues at different stages of diseases severity. J. Bacteriol. Virol..

[9] Santimaleeworagun W, Changpradub D, Thunyaharn S, Hemapanpairoa J (2019). Optimizing the dosing regimens of daptomycin based on the susceptible dose-dependent breakpoint against vancomycin-resistant enterococci infection. Antibiotics.

[10] Roach DR, Donovan DM (2015). Antimicrobial bacteriophage-derived proteins and therapeutic applications. Bacteriophage.

[11] Ha E, Son B, Ryu S (2018). *Clostridium perfringens* virulent bacteriophage CPS2 and its thermostable endolysin LysCPS2. Viruses.

[12] Wang S, Gu J, Lv M, Guo Z, Yan G, Yu L (2017). The antibacterial activity of *E. coli* bacteriophage lysin lysep3 is enhanced by fusing the *Bacillus amyloliquefaciens* bacteriophage endolysin binding domain D8 to the C-terminal region. J. Microbiol..

[13] Love MJ, Coombes D, Ismail S, Billington C, Dobson RCJ (2022). The structure and function of modular *Escherichia coli* O157:H7 bacteriophage FTBEc1 endolysin, LysT84: defining a new endolysin catalytic subfamily. Biochem. J..

[14] Park Y, Lim JA, Kong M, Ryu S, Rhee S (2014). Structure of bacteriophage SPN1S endolysin reveals an unusual two-module fold for the peptidoglycan lytic and binding activity. Mol. Microbiol..

[15] Kong M, Na H, Ha NC, Ryu S (2019). LysPBC2, a novel endolysin harboring a *Bacillus cereus* spore binding domain. Appl. Environ. Microbiol..

[16] Schmelcher M, Shabarova T, Eugster MR, Eichenseher F, Tchang VS, Banz M (2010). Rapid multiplex detection and differentiation of Listeria cells by use of fluorescent phage endolysin cell wall binding domains. Appl. Environ. Microbiol..

[17] Adriaenssens EM, Brister JR (2017). How to name and classify your phage: an informal guide. Viruses.

[18] Kang HY, Kim S, Kim J (2015). Isolation and characterization of an *Enterococcus faecalis* bacteriophage. Kor. J. Microbiol..

[19] Uchiyama J, Rashel M, Maeda Y, Takemura I, Sugihara S, Akechi K (2008). Isolation and characterization of a novel *Enterococcus faecalis* bacteriophage phiEF24C as a therapeutic candidate. FEMS Microbiol. Lett..

[20] Xu X, Zhang D, Zhou B, Zhen X, Ouyang S (2021). Structural and biochemical analyses of the tetrameric cell binding domain of Lys170 from enterococcal phage F170/08. Eur. Biophys J..

[21] Proença D, Velours C, Leandro C, Garcia M, Pimentel M, São-José CA (2015). A two-component, multimeric endolysin encoded by a single gene. Mol. Microbiol..

[22] Kim S, Lee DW, Jin JS, Kim J (2020). Antimicrobial activity of LysSS, a novel phage endolysin, against *Acinetobacter baumannii* and *Pseudomonas aeruginosa*. J. Glob. Antimicrob. Res..

[23] Kelley LA, Mezulis S, Yates CM, Wass MN, Sternberg MJ (2015). The Phyre2 web portal for protein modeling, prediction and analysis. Nat. Protoc..

[24] Bae M, Park J, Seong H, Lee H, Choi W, Noh J (2022). Rapid extraction of viral nucleic acids using rotating blade lysis and magnetic beads. Diagnostics.

[25] Kretzer JW, Lehmann R, Schmelcher M, Banz M, Kim KP, Korn C (2007). Use of high-affinity cell wall-binding domains of bacteriophage endolysins for immobilization and separation of bacterial cells. Appl. Environ. Microbiol..

[26] Rahman MU, Wang W, Sun Q, Shah JA, Li C, Sun Y (2021). Endolysin, a promising solution against antimicrobial resistance. Antibiotics.

[27] Kunstmann S, Scheidt T, Buchwald S, Helm A, Mulard LA, Fruth A (2018). Bacteriophage Sf6 tailspike protein for detection of *Shigella flexneri* pathogens. Viruses.

[28] Eugster MR, Haug MC, Huwiler SG, Loessner MJ (2011). The cell wall binding domain of Listeria bacteriophage endolysin PlyP35 recognizes terminal GlcNAc residues in cell wall teichoic acid. Mol. Microbiol..

[29] Kong M, Shin JH, Heu S, Park JK, Ryu S (2017). Lateral flow assay-based bacterial detection using engineered cell wall binding domains of a phage endolysin. Biosens. Bioelectron..

[30] Garde S, Calzada J, Sánchez C, Gaya P, Narbad A, Meijers R (2020). Effect of *Lactococcus lactis* expressing phage endolysin on the late blowing defect of cheese caused by *Clostridium tyrobutyricum*. Int. J. Food Microbiol..

[31] Ma H, Ó'Fágáin C, O'Kennedy R (2020). Antibody stability: a key to performance - analysis, influences and improvement. Biochimie.

[32] Langley G, Besser J, Iwamoto M, Lessa FC, Cronquist A, Skoff TH (2015). Effect of culture-independent diagnostic tests on future emerging infections program surveillance. Emerg. Infect. Dis..

[33] Ha SM, Kim CK, Roh J, Byun JH, Yang SJ, Choi SB (2019). Application of the whole genome-based bacterial identification system, TrueBac ID, using clinical isolates that were not identified with three matrix-assisted laser desorption/ionization time-offlight mass spectrometry (MALDI-TOF MS) systems. Ann. Lab. Med..

[34] da Silva DAV, Brendebach H, Grützke J, Dieckmann R, Soares RM, de Lima JTR (2020). MALDI-TOF MS and genomic analysis can make the difference in the clarification of canine brucellosis outbreaks. Sci. Rep..

[35] Law JW, Ab Mutalib NS, Chan KG, Lee LH (2014). Rapid methods for the detection of foodborne bacterial pathogens: principles, applications, advantages and limitations. Front. Microbiol..

[36] Giuliano C, Patel CR, Kale-Pradhan PB (2019). A guide to bacterial culture identification and results interpretation. P T.

[37] Mancini N, Carletti S, Ghidoli N, Cichero P, Burioni R, Clementi M (2010). The era of molecular and other non-culture-based methods in diagnosis of sepsis. Clin. Microbiol. Rev..

[38] Bhattacharya S (2013). Early diagnosis of resistant pathogens: how can it improve antimicrobial treatment?. Virulence.

[39] Batra A, Cottam D, Lepesteur M, Dexter C, Zuccala K, Martino C (2023). Development of a rapid, low-cost portable detection assay for enterococci in wastewater and environmental waters. Microorganisms.

[40] Zhu B, Hu J, Li X, Li X, Wang L, Fan S (2022). Rapid and specific detection of *Enterococcus faecalis* with a visualized isothermal amplification method. Front. Cell. Infect. Microbiol..

[41] Dreier M, Berthoud H, Shani N, Wechsler D, Junier P (2020). SpeciesPrimer: a bioinformatics pipeline dedicated to the design of qPCR primers for the quantification of bacterial species. Peer J..

[42] Gigante AM, Olivença F, Catalão MJ, Leandro P, Moniz-Pereira J, Filipe SR (2021). The mycobacteriophage Ms6 LysB Nterminus displays peptidoglycan binding affinity. Viruses.

[43] Muhammad Jai HS, Dam LC, Tay LS, Koh JJW, Loo HL, Kline KA (2020). Engineered lysins with customized lytic activities against enterococci and staphylococci. Front. Microbiol..

[44] Regulski K, Courtin P, Kulakauskas S, Chapot-Chartier MP (2013). A novel type of peptidoglycan-binding domain highly specific for amidated D-Asp cross-bridge, identified in *Lactobacillus casei* bacteriophage endolysins. J. Biol. Chem..

