# Supplementary figures and images for: Development of a Magnetic Bead-Based Method for Specific Detection of Enterococcus faecalis Using C-Terminal Domain of ECP3 Phage Endolysin
Source: J Microbiol Biotechnol. 2023 Apr 28;33(7):964–72. doi: 10.4014/jmb.2302.02033 (PMC10394340; doi:10.4014/jmb.2302.02033)

Supplementary Fig. 1.

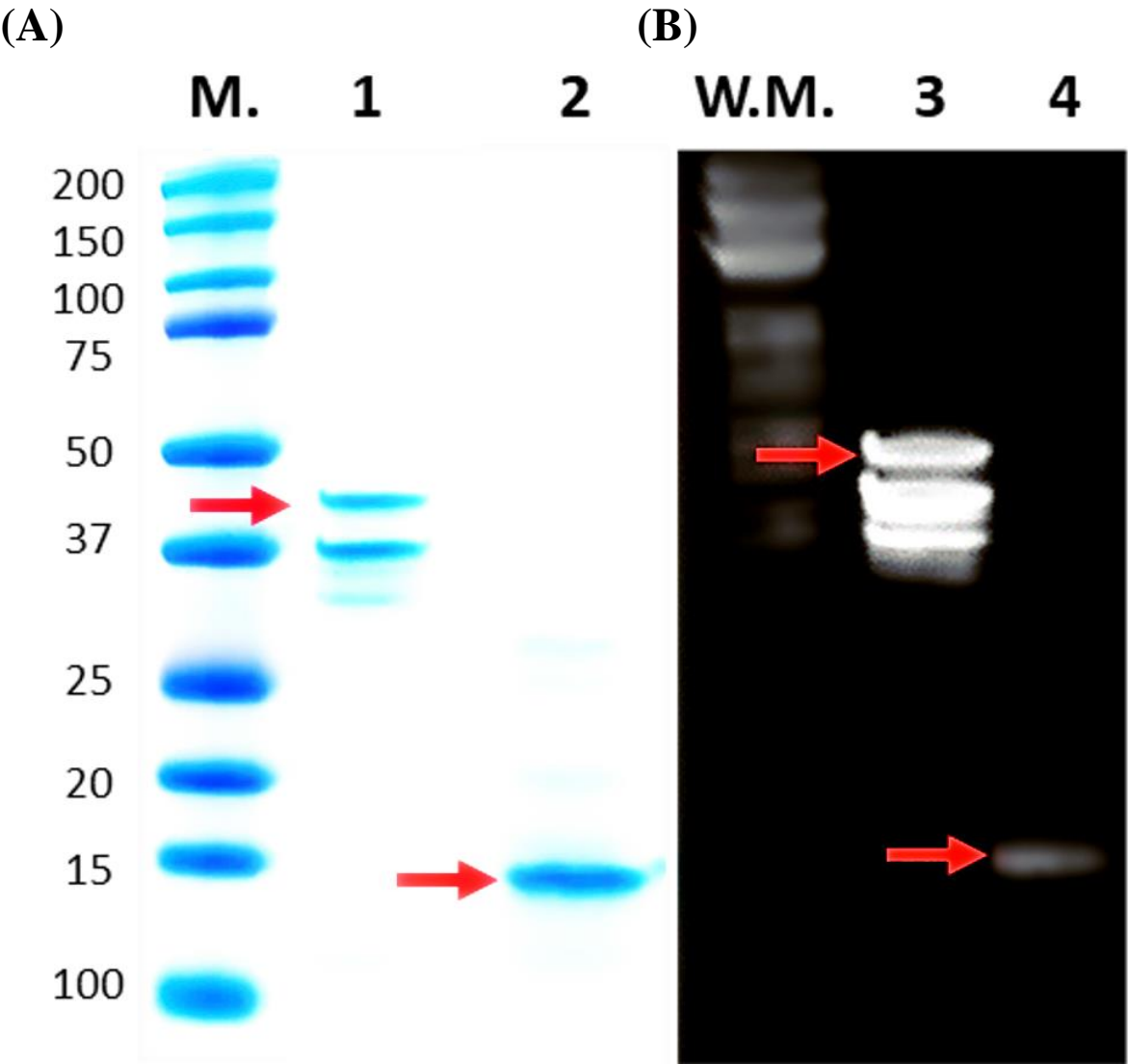

Supplementary Fig. 2.

(A)

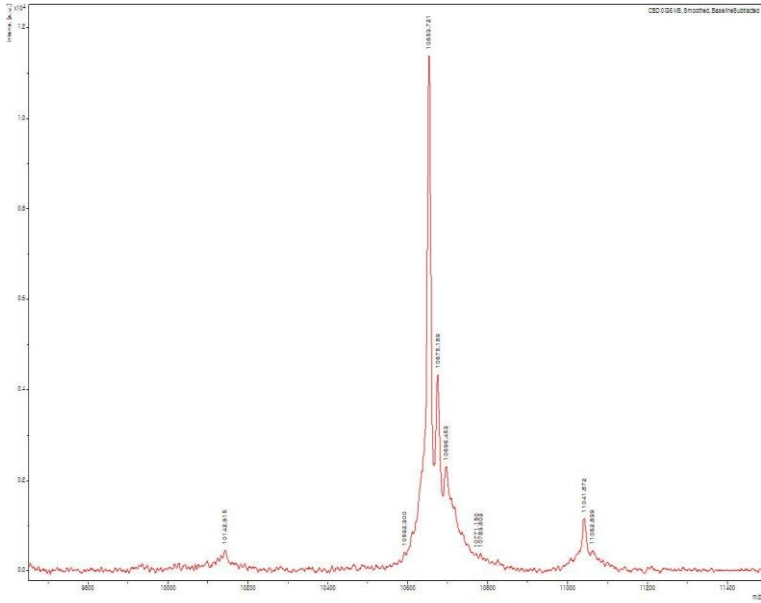

(B)

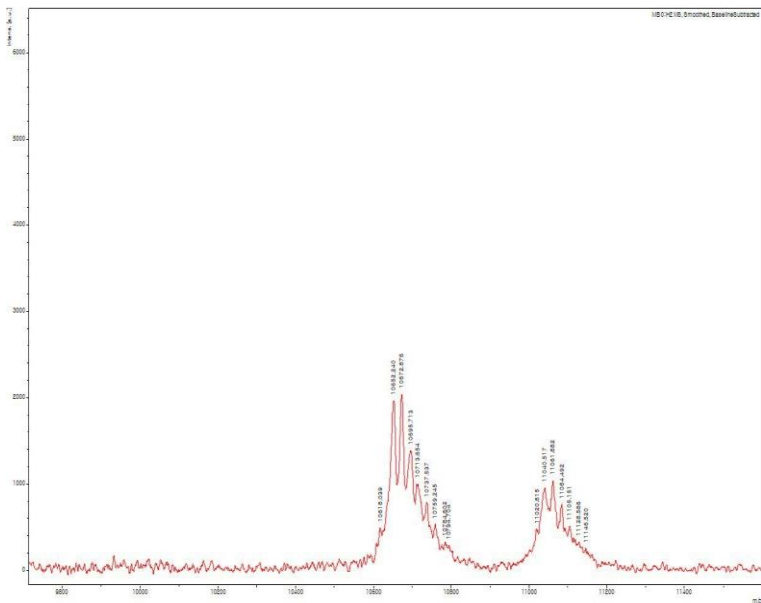

(C)

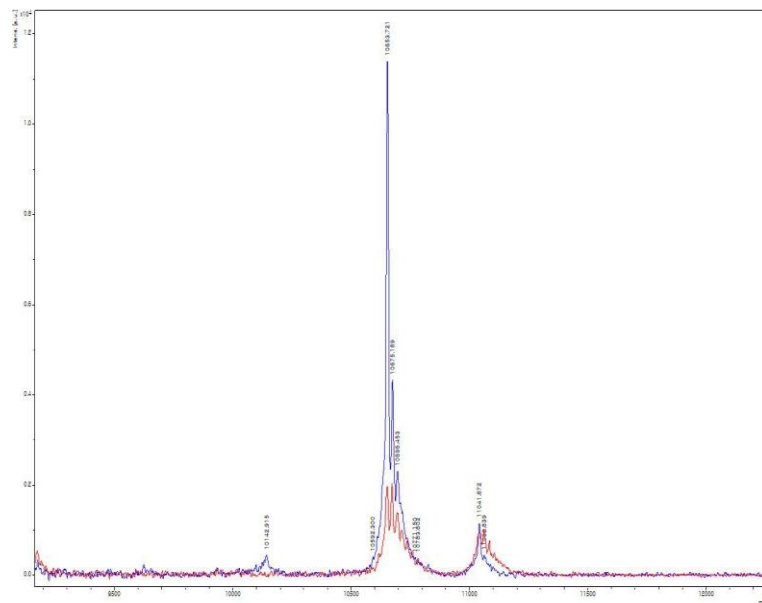

Supplement: Supplementary file 1 [file jmb-33-7-964-supple.pdf]
